# Ectopic cervical thymoma: a rare entity in the anterior neck

**DOI:** 10.1186/s13019-024-02808-6

**Published:** 2024-06-04

**Authors:** Yang Yang, Cheng Shen, Lin Ma

**Affiliations:** 1 Department of Thoracic Surgery, West-China Hospital, Sichuan University, Chengdu, 610041 China; 2 Department of Thoracic Surgery, Lanzhou Petrochemical General Hospital (The Fourth Affiliated Hospital of Gansu University of Chinese Medicine), Lanzhou, 730060 China

**Keywords:** Ectopic cervical thymoma, Surgery, Pathology

## Abstract

Thymoma is a rare malignancy with usual location in the antero-superior mediastinum. Ectopic cervical thymoma (ECT) is an extremely rare tumor that originates from ectopic tissue, and is caused by the aberrant migration of the embryonic thymus. Our patient was a 56-year-old man who had a nodular lesion in the neck for several years. Computed tomography and Enhanced magnetic resonance imaging were performed. He underwent surgery, and a histological examination resulted in a diagnosis of type AB thymoma.

During a regular health check, a cervical mass was detected on the chest radiography of a 56-year-old man. He did not report any symptoms such as chest pain, hoarseness, hemoptysis, coughing, difficulty breathing or myasthenia gravis symptoms. The patient was a non-smoker and had no history of exposure to environmental fumes or dust. Physical examination showed normal breath sounds in both lung fields. Thyroid function indicators were also within normal limits. Hematology and biochemistry tests were within the normal range. The patient was then admitted to the hospital for further examination.

Plain and contrast-enhanced neck and chest computed tomography (CT) (Fig. 1A and B) revealed irregular mass shadow was observed in the anterior neck, posterior to the sternum, which protruded noticeably from the surface. The mass had a relatively large cross-sectional area of approximately 6.3*4.6 cm. It showed mild and uniform enhancement, with unclear demarcation between the local area and the posterior thyroid. Enhanced Magnetic Resonance Imaging (MRI) (Fig. 1C and D) of neck and chest showed that the mass with T1 iso-intensity and T2 high-intensity signals was observed in the anterior neck and upper anterior mediastinum, posterior to the sternum. On diffusion weighted imaging (DWI) in MRI, it showed high-intensity signals and had a size of approximately 6.8 × 4.8 × 6.5 cm. The mass showed uneven enhancement and contained small patchy short-T1 and long-T2 signal shadows. The boundary between the lesion area and the posterior thyroid and left jugular vein wall was not clear. Additionally, there was an increase in the number and size of lymph nodes in both the left supraclavicular fossa and mediastinum.

**Fig. 1:**
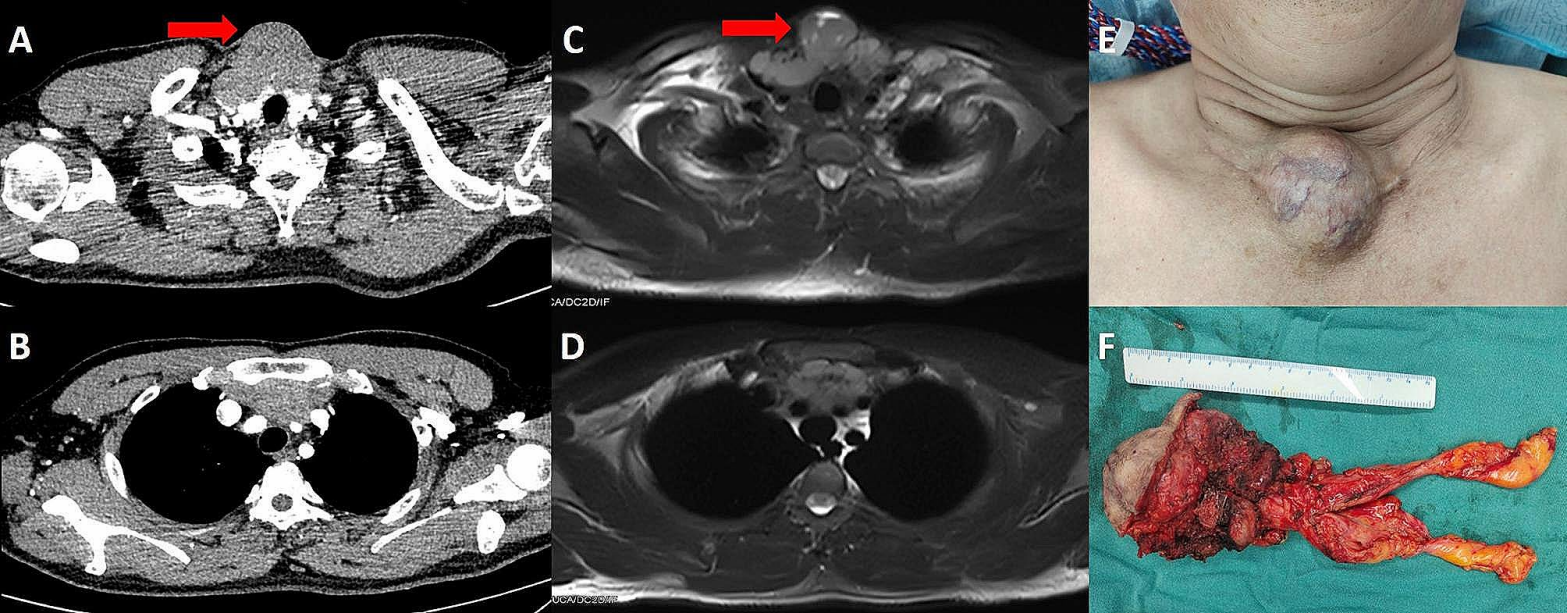
**A and B**: Plain and contrast-enhanced neck and chest CT revealed irregular mass shadow was observed in the anterior neck, posterior to the sternum, which protruded noticeably from the surface. The mass had a relatively large cross-sectional area of approximately 6.3*4.6 cm. **C and D**: Enhanced MRI of neck and chest showed that the mass with T1 iso-intensity and T2 high-intensity signals was observed in the anterior neck and upper anterior mediastinum, posterior to the sternum. On DWI, it showed high-intensity signals and had a size of approximately 6.8 × 4.8 × 6.5 cm. The mass showed uneven enhancement and contained small patchy short-T1 and long-T2 signal shadows. **E and F**: A mass was observed in the anterior neck and it was resected completely

A mass was observed in the anterior neck (Fig. 1E), but the patient refused to undergo fine needle biopsy. As diagnosis of the mass in the thorax was not established through imaging, Ectopic cervical thymoma resection and thymectomy were scheduled. The sternum saw split open the sternum, and bone wax hemostasis was explored. The tumor was partially excised from the skin of the anterior part of the neck to the bilateral sternoclavicular joint and bilateral thyroid gland. It was found that the bilateral sternoclavicular joint was partially affected. The left unnamed vein was particularly affected. Thyroid surgery was requested to assist in exposing the left internal jugular vein and left common carotid artery, and monitoring the vagus nerve was performed. Gradually, the small blood vessels were clamped with titanium clips and synthetic clips, exploring along the left internal jugular vein to the left innominate vein. After being blocked by forceps, the affected part of the left innominate vein was continuously sutured and severed. It was found that the lower pole of the right thyroid was affected, and a thyroid surgeon assisted in removing the affected part of the right lobe of the thyroid. The right internal jugular vein and right common carotid artery were also exposed, and the right innominate vein continued to swim downwards. It was found that the right innominate vein was not affected, and the tumor and bilateral sternoclavicular joints were completely removed along the upper part of the right innominate vein resection of pre pericardial fat and mediastinal lymph node dissection was performed, with stations 2, 3, 4, 5 and 6 lymph nodes removed. (Fig. 1F). After the surgery, the patient was transferred to the intensive care unit and moved to general care after 24 h. Within one week, normal food intake was renovated and the patient was discharged from the hospital. Based on the histopathological findings of lymphocyte-poor spindle cells and lymphocyte-rich polygonal cells, the tumor was identified as a type AB thymoma according to the 2004 World Health Organization (WHO) histological classification. (Fig. 2). One month later, a chest CT scan showed the residual lobe could finally be fully expanded to an almost standard size and appearance. The patient is still alive and is currently receiving follow-up.

**Fig. 2:**
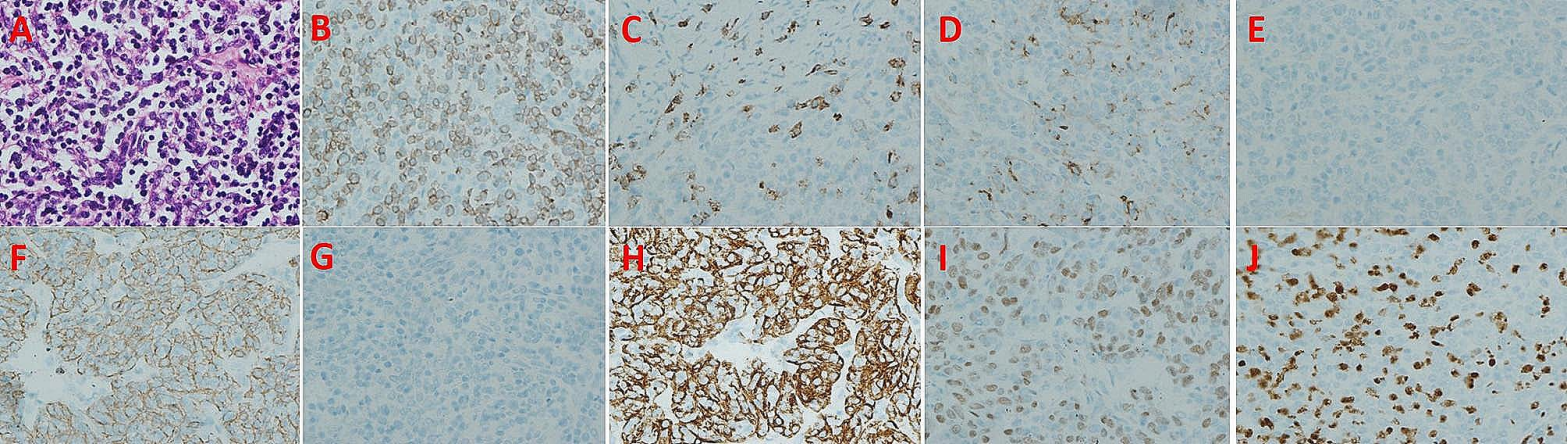
Histopathological findings show that the tumor was lymphocyte-poor spindle cells and lymphocyte-rich polygonal cells, which was identified as a type AB thymoma according to the 2004 WHO histological classification. **A**: Hematoxylin-eosin staining, **B**: CD3, **C**: CD5, **D**: CD20, **E**: CD117, **F**: CK19, **G**: CK20, **H**: CKP, **I**: P63, **J**: TDT

## Discussion

Compared to thymomas found in the mediastinum, thymomas originating from ectopic cervical thymic tissue are uncommon and typically affect females with a median age of 53.5 years (range, 32–86 years) [1]. The diagnosis of ectopic cervical thymomas (ECT) can be challenging due to inconsistent clinical presentations and the prevalence of more common pathologies in this anatomical site [1, 2]. ECT may be misidentified as thyroid cancer or lymphoma. The primary symptom of thymomas is often a palpable mass near the thyroid gland, and patients with thymomas may experience symptoms such as pain, respiratory insufficiency, or superior vena cava syndrome resulting from complications caused by local compression [2].

The CT and MRI scans indicated that the tumor had infiltrated the surrounding tissues, such as the sternocleidomastoid muscle and the internal carotid vein. CT scan results typically show a homogeneous mass closely adherent to the carotid sheath in cases of an ectopic thymic mass. This tumor can exert pressure on nearby neck structures, such as the cervical trachea or blood vessels, resulting in a mass effect. However, the patient’s impaired renal function prevented the use of contrast material, making a thorough evaluation through diagnostic imaging impossible. When diagnosing thymomas in the neck, the presence of a septal structure within the tumor as seen on T2-weighted MRI scans is a crucial imaging feature to consider [3]. MRI can aid in diagnosing ectopic cervical thymus by providing clear tissue plane delineation, often showing a connection between the cervical mass and the mediastinal thymus or indicating similarity in density to normal thymic tissue. Scintigraphy using thallium-201 or technetium-99, as well as radioactive iodine, can assist in the differential diagnosis of ECT [4].

Due to its low frequency, diagnosing ECT can be challenging, as its histological characteristics are similar to mediastinal/thymic thymomas. Fine needle aspiration cytology or frozen section examination may lead to a diagnostic pitfall, as it could suggest a lymphomatous process or undifferentiated carcinoma, depending on the predominance of lymphocytes and inconspicuous epithelial cells [5, 6]. Immunohistochemistry remains crucial for tissue diagnosis, while cytology is often less useful. Thymoma is primarily composed of thymic epithelial tumors, with varying amounts of lymphoid tissue. Although other tumors like lymphomas and germ cell tumors can develop in the thymus, thymomas and thymic carcinomas specifically arise from true thymus elements. Histopathologically, thymomas exhibit a range of features including epidermoid cells, squamous cells, spindle cells, papillary lesions, and degenerated Hassall’s corpuscles. Thymic lymphomas, on the other hand, manifest as thymus gland enlargement due to lymphocytic proliferation, which replaces the normal thymus architecture with a population of lymphoblasts.

The preferred treatment for patients with ectopic cervical thymoma is surgical resection. Patients with involvement of neck lymph nodes or extrathyroidal extension may be considered for adjuvant radiation therapy. In cases where there is capsular invasion in ectopic thymomas, it is crucial to administer adjuvant radiotherapy to decrease the likelihood of local recurrence, similar to the approach taken with mediastinal thymomas [5].

## Data Availability

No datasets were generated or analysed during the current study.
